# Evolution of the SARS-CoV-2 spike protein in the human host

**DOI:** 10.1038/s41467-022-28768-w

**Published:** 2022-03-04

**Authors:** Antoni G. Wrobel, Donald J. Benton, Chloë Roustan, Annabel Borg, Saira Hussain, Stephen R. Martin, Peter B. Rosenthal, John J. Skehel, Steven J. Gamblin

**Affiliations:** 1 Structural Biology of Disease Processes Laboratory, NW1 1AT London, UK; 2 Structural Biology Science Technology Platform, NW1 1AT London, UK; 3 Worldwide Influenza Centre, NW1 1AT London, UK; 4 RNA Virus Replication Laboratory, NW1 1AT London, UK; 5 Structural Biology of Cells and Viruses Laboratory; Francis Crick Institute, NW1 1AT London, UK

**Keywords:** Cryoelectron microscopy, SARS-CoV-2, Viral evolution

## Abstract

Recently emerged variants of SARS-CoV-2 contain in their surface spike glycoproteins multiple substitutions associated with increased transmission and resistance to neutralising antibodies. We have examined the structure and receptor binding properties of spike proteins from the B.1.1.7 (Alpha) and B.1.351 (Beta) variants to better understand the evolution of the virus in humans. Spikes of both variants have the same mutation, N501Y, in the receptor-binding domains. This substitution confers tighter ACE2 binding, dependent on the common earlier substitution, D614G. Each variant spike has acquired other key changes in structure that likely impact virus pathogenesis. The spike from the Alpha variant is more stable against disruption upon binding ACE2 receptor than all other spikes studied. This feature is linked to the acquisition of a more basic substitution at the S1-S2 furin site (also observed for the variants of concern Delta, Kappa, and Omicron) which allows for near-complete cleavage. In the Beta variant spike, the presence of a new substitution, K417N (also observed in the Omicron variant), in combination with the D614G, stabilises a more open spike trimer, a conformation required for receptor binding. Our observations suggest ways these viruses have evolved to achieve greater transmissibility in humans.

## Abstract (web summary)

The SARS-CoV-2 spike has been evolving in the human population. The variants of concern alpha and beta evolved to optimise spike openness and so ability to bind its receptor ACE2, the affinity towards the receptor, and stability upon receptor binding.

## Introduction

The SARS-CoV-2 spike glycoprotein is the major surface antigen of the virus. Its function is to bind the host receptor ACE2 and mediate the subsequent membrane fusion required for cell entry^1–8^. The virus has evolved in the human host during the pandemic^9–11^ and we and others have demonstrated that the predominant D614G substitution, located in a monomer-monomer interface of the spike trimer, increases its propensity to adopt the open conformation that is competent to bind receptor^12–14^. The D614G substitution has been shown to decrease shedding of S1 from spike on virions, consistent with increased stability of the pre-fusion conformation^13,15^. Recently emerging variants of SARS-CoV-2 have acquired other substitutions in the spike including a number located at the monomer-monomer interfaces, at the receptor-binding site, and near the furin-cleavage site (Supplementary Fig. 1). Here we have examined the structures and receptor-binding properties of spikes of the B.1.1.7 variant first described in Kent, United Kingdom (now termed Alpha)^10,16–19^ and the B.1.351 variant first described in South Africa (now termed Beta)^20,21^. We used pre-fusion-stabilised spikes^22^ which have been established as a tool to study the receptor-binding properties in the absence of the conformational change associated with fusion. This enabled us to directly compare the pre-fusion spikes of the new variants with those of the original strain (first identified in Wuhan)^5,8^ and the D614G-only variant^12^ we described in previous studies, findings that agree with reports that used non-stabilised spikes^6,7,13,23^.

Two recent studies showed increased RBD erectability and enhanced receptor binding by the Alpha and Beta spikes^24,25^. Our findings confirm and extend these observations, providing insights into their structures with bound receptor; and revealing several characteristics that these variant spikes evolved to optimise their interaction with the host, which could explain their increased infectivity.

## Results and discussion

**Fig. 1:**
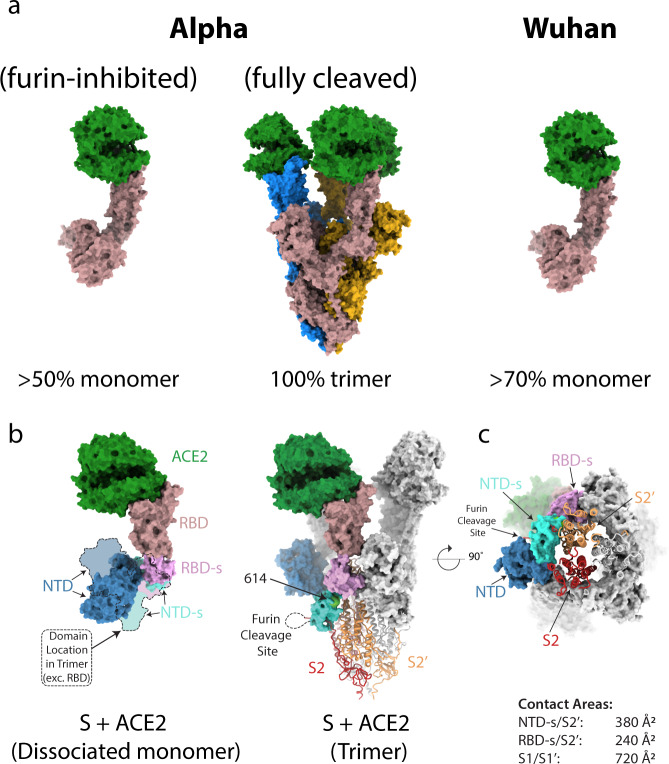
Structures of Alpha variant spike interacting with ACE2 receptor. **a** Surface representations of predominant molecular species present when ACE2 is mixed with the Alpha variant spike expressed in the presence (left panel) or absence (middle panel) of furin inhibitor I, compared to the predominant species when ACE2 is mixed with the original (Wuhan) spike from our previous study (right panel) (PDB ID 7A91^5^). ACE2 is coloured in green, with monomers of the spike coloured in blue, goldenrod, and rosy brown in the middle panel and rosy brown for S monomers in the right and left panels. **b** Comparison of the domain movements of S complexed to ACE2 in its trimeric (middle) compared to monomeric (left) form. The monomeric and trimeric ACE2 S1 subunits can be aligned very closely on the ACE2/RBD components but the NTD subdomain (NTD-s, pallid blue) and the NTD (blue) are rotated by 95**°** and their centre of mass translated by 25 Å in the monomer compared with the trimer, resulting in a conformation incompatible with maintenance of the trimeric state. The furin-cleavage site lies on a loop (shown as dotted line) between the C-terminus of the NTDG and S2 and thus cleavage appears to be able to release sufficient strain to allow the resulting ACE2 complex to remain stable as a trimer. The S1 subunits are shown as surface representation and the S2 core as cartoons. **c** The ring formed by S1 subunits of the trimer upon binding to three ACE2 molecules (surface representation) remains attached to the exposed S2 core through interactions between the S2′ subunit of one monomer (peach) and NTD-s (pallid blue) and RBD-s (plum) domains of the adjacent one. Domains are coloured: RBD in rosy brown, RBD subdomain (RBD-s) in plum, NTD subdomain (NTD-s) in cyan, NTD in navy, and S2 of the same chain as coloured S1 domains in red.

First, examination of the 2D class averages (Supplementary Fig. 2) of ACE2-bound Alpha spike with its furin site intact reveals that all spikes in this dataset are present as trimers (Fig. 1a, Supplementary Table 1). This is the first cleaved SARS-CoV-2 spike protein in complex with ACE2 we have observed to remain fully trimeric upon receptor binding. A substantial proportion of all other spike/ACE2 complexes are dissociated into monomers^5^. For example, in our previous study of the furin-cleaved spike of the original Wuhan strain (Wuhan) complexed with ACE2 (Fig. 1a), we observed that more than 70% of the particles present were monomeric S1-S2/ACE2 complexes^5^. There are many factors that prohibit quantitative estimates of solution equilibria from electron micrographs. However, given that the present study was done under the same conditions as our earlier studies, it is reasonable to conclude that the trimeric Alpha spike is more stable in complex with the receptor than the Wuhan^5^ or Beta spikes (this study). We also observed that the Alpha variant spike is almost fully cleaved into S1 and S2 (Supplementary Fig. 3a), which was not observed for the Wuhan spike^7,8^. This observation is consistent with one of the changes in the Alpha spike being the substitution P681H, which generates an even more basic furin-cleavage site (HRRAR). A similar observation has been made in studies of spike material isolated directly from the Alpha variant virus^26^ and the same P681H substitution has also been observed in the recent B.1.1.529 (Omicron) variant spike.

To test if the cleavage of the S1-S2 subunits is directly responsible for the greater stability of the trimeric ACE2-bound S1/S2 complex, we expressed the Alpha spike in the presence of a furin inhibitor, decanoyl-Arg-Val-Lys-Arg-chloromethylketone, which resulted in an almost completely uncleaved protein (Supplementary Fig. 3b). Incubation of this uncleaved spike with ACE2 resulted in more than 50% of particles being monomeric S1-S2/ACE2 (Fig. 1a, Supplementary Fig. 2). This conformation, like that of the Wuhan strain S1-S2/ACE2 complex, is incompatible with the trimeric structure (Fig. 1b). This might be the result of some local effect not immediately evident, such as the recently reported allosteric effects of ACE2 binding on the local dynamics of the S1-S2 linker^27^, or a consequence of the (disordered) S2 moiety carried along with the dissociated S1:ACE2 from the uncleaved protein, since preponderance of monomer S/ACE2 species in other SARS-CoV-2 variants, such as Wuhan (Fig. 1a) and Beta (Supplementary Fig. 4), correlates with the lower levels of cleavage of these spikes (Supplementary Fig. 3). In addition our structures of unbound, uncleaved Alpha spike (Supplementary Figs. 5 and 6) suggest the trimeric state of the receptor-bound form of the Alpha variant spike might be further stabilised by the substitutions D1118H and A570D on the inter-monomer interfaces (Supplementary Fig. 5), as also recently suggested by Yang et al.^28^.

**Fig. 2:**
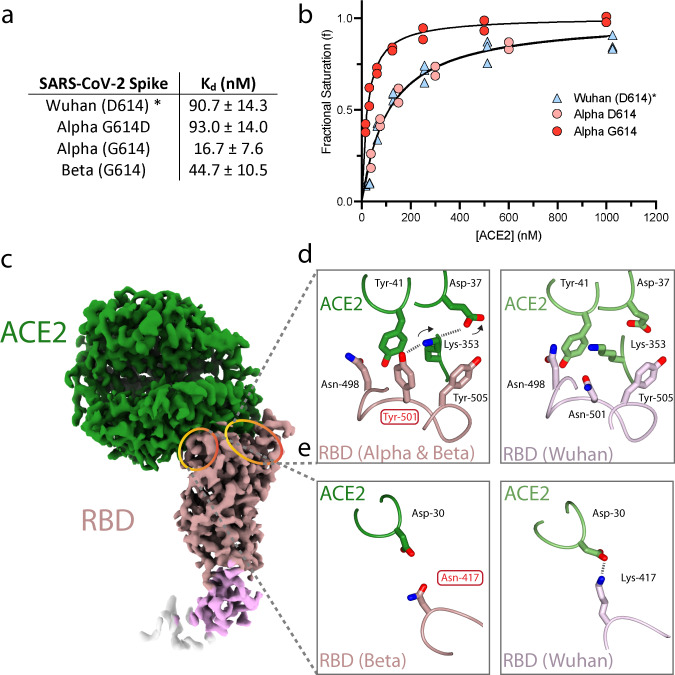
Variant spike binding to receptor ACE2. **a** *K*_d_ of variant spikes binding to ACE2 measured using biolayer interferometry and calculated from *k*_off_/*k*_on_ analysis (see Supplementary Fig. 7 for details). **b** Plots of fractional saturation binding measurements with data for the Alpha variant shown in red, Alpha variant with G614D substitution in pink, and Wuhan (D614) spike in blue. Wuhan (*the data for which shown here are adapted from our previous work^47^) and Alpha D614 spike show almost identical affinity towards ACE2. Similar results were obtained for G614 vs D614 mink (Y453F) spike (Supplementary Figs. 4 and 7). **c** cryoEM density of the complex of Beta variant S1 with ACE2, with ACE2 coloured in green, RBD in rosy brown, RBD subdomain in plum, with the remaining S1 disseminated density in cream. **d**, **e** Detail of changes in the binding interfaces present in variants (left column) compared to the Wuhan strain (right column). **d** The N501Y substitution present in both the Beta and Alpha variants allows formation of a new hydrogen bond or a salt bridge. **e** The K417N substitution present in the Beta variant eliminates a salt bridge between the RBD and ACE2. Source data are provided as a Source Data file.

Second, in line with a recent study by Cai et al.^24^, we found enhanced binding of ACE2 to Alpha and Beta variant spikes when compared to Wuhan by surface biolayer interferometry. The data (Fig. 2a, b; Supplementary Fig. 7) show a sixfold increase in binding strength for Alpha spike, and a twofold increase for Beta spike (compared to Wuhan) arising from the shared substitution N501Y in the RBD. The substitution of the asparagine at position 501 in Wuhan for a tyrosine residue in both Alpha & Beta variants (Fig. 2c) leads to an increase in hydrophobic interactions between the aromatic ring of Y501_(RBD)_ and the aromatic ring of Tyr-41_(ACE2)_ and the aliphatic moiety of Lys-353_(ACE2)_, in addition to a charged hydrogen bond between the phenolic hydroxyl of Tyr-501_(RBD)_ and Lys-353_(ACE2)_ (Fig. 2d). The smaller increase in affinity for ACE2 of the Beta spike versus Alpha spike, is consistent with the finding that, whereas Alpha has retained the same salt bridge as Wuhan between Lys-417_(RBD)_ and Asp-30_(ACE2)_, the RBD of Beta has acquired the additional substitution of an asparagine at residue 417 which cannot make the equivalent salt bridge (Fig. 2e).

To understand the evolution of receptor binding, we also expressed Alpha spike with an aspartic acid, rather than glycine residue, at position 614. The substitution D614G (relative to Wuhan) occurred earlier in the evolution of SARS-CoV-2, became the predominant global form of the virus^9^ and continues to be present in the Alpha and Beta variant forms of the virus. The engineered G614D Alpha spike (Y501, D614) shows the same binding affinity as Wuhan (N501, D614) (Fig. 2a, b; Supplementary Fig. 7). Similarly, we also show that the Y453F substitution in the RBD of mink spike, a structure of which we also report here (Supplementary Figures 4–6), only increases affinity for human ACE2 if residue 614 is a glycine but not if it is an aspartic acid (Supplementary Fig. 7).

**Fig. 3:**
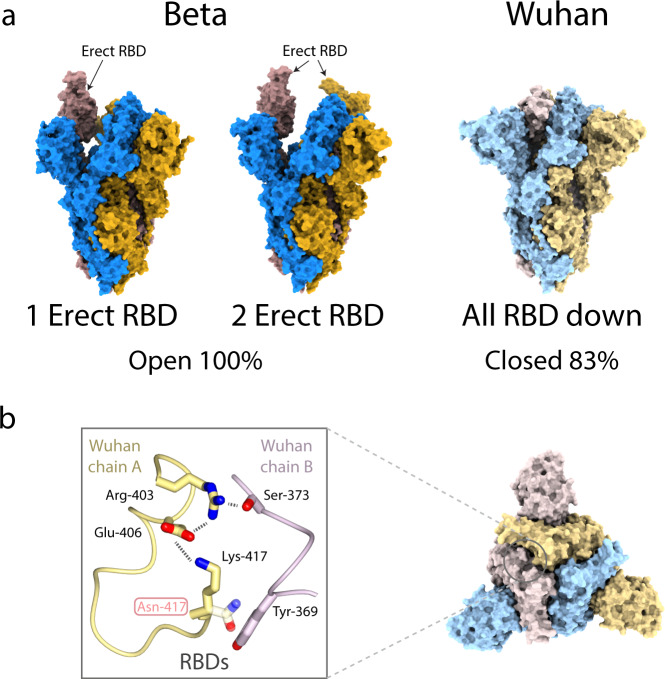
Beta variant spike trimer structures. **a** (Left and centre) Surface representations of cryoEM structures of the Beta variant spike, with monomers coloured in blue, goldenrod and rosy brown, compared to (right) the closed form of the Wuhan strain determined in our previous study (PDB ID 6ZGE^8^) shown in paler hues of the same colours. The Beta variant adopts only open conformations with either one (64%) or two (36%) of the RBDs in the erect, receptor-binding-competent form. **b** The K417N substitution present in the Beta variant likely destabilises the closed form of the protein by introducing a steric clash and interfering with the network of electrostatic interactions present at the trimer interface in the closed form of the Wuhan protein. Strongly charged R417 in the fully-closed Pangolin-CoV spike seems to stabilise the same network of RBD-RBD interactions^47^.

Third, the most striking feature of the structure of Beta spike revealed by cryoEM is that all the trimers adopt an open conformation. This is in contrast to our earlier study, under the same conditions, that showed 83% of Wuhan spike particles were in the closed form (Fig. 3a)^8^. Again, solution equilibria cannot be extracted from these data but we interpret this near-complete switch in states as indicative of the open form of the Beta variant being more stable than the open form of Wuhan. Two recent studies also report Beta spike showing a higher proportion of open conformation relative to earlier viruses^24,25^. Inspection of the sequence of the Beta spike and comparison of its structure with that of the closed form of Wuhan spike and the open and closed forms of G614 spikes, suggests that the opening of Beta spike could be driven by the substitution K417N on the background of G614 (both also observed in the Omicron variant spike). In Wuhan, Lysine at residue 417 on the RBD not only makes an aliphatic packing interaction with Tyr-369 of the neighbouring subunit, it also forms a salt bridge/hydrogen bond network with Glu-406 and Arg-403 of the RBD and with Ser-373 of the neighbouring RBD that stabilises the closed conformation. In contrast, the substitution of an asparagine at position 417 in Beta removes an intramolecular salt bridge and would generate a steric clash with Tyr-369 of the neighbouring RBD leading to destabilisation of the closed form and thus promotion of the open form (Fig. 3b). In the same way that G614 is a prerequisite for realising tighter receptor binding by the substitution N501Y described above, it may also enable spike protein from the Beta virus to achieve an open conformation as a result of the K417N substitution.

We have shown that the almost fully cleaved Alpha spike is more stable as a receptor-bound trimer than the Wuhan or Beta spikes which are subject to less efficient cleavage. We cannot explain why near-complete cleavage produces more stable spike/ACE2 complexes but similar increases in stability have been observed following cleavage of the influenza haemagglutinin precursor^29,30^ and we suggest that such enhanced stability increases the likelihood of productive membrane fusion events. The recent variants of concern B.1.617 (Kappa) and B.1.617.2 (Delta)^31–33^ contain the substitution P681R, which also results in full cleavage (Supplementary Fig. 3c). Our data suggest that this substitution P681R in Kappa and Delta spikes, as well as P681H in Omicron spike, will also increase stability of the receptor-bound form of these spikes, accounting at least in part for their increased transmissibility. Our binding data on the variants and engineered constructs show that the D614G substitution is a prerequisite for the tighter receptor binding of changes in RBD, like N501Y, but do not explain how it facilitates the increase in affinity. We suggest that one of effects of the more open conformation promoted by the D614G substitution is to increase the proportion of accessible RBD-binding sites, thus enhancing the avidity of virus binding to host cells.

We have demonstrated that the emergence of Alpha spike, which is completely cleaved, and Beta spike, which we have not observed to adopt the closed conformation, represent two related recent steps in viral adaptation to the human host. They follow D614G substitution which was acquired early in the pandemic and similarly acted to increase the spike stability^12–15^. We suggest that modifications in the spike glycoprotein during evolution of the SARS-CoV-2 virus in humans may have made the virus more infectious by promotion of the open forms of spike, increasing the stability of the pre-fusion-conformation of the receptor-bound trimeric spike, and by tighter receptor binding.

## Methods

### Construct design

The SARS-CoV-2 spike constructs used in this study were derived by Genscript from the spike ectodomain (residues 1-1208) constructs of the Wuhan variant cloned into pcDNA.3.1(+) described before^5,8^. The variant spikes were stabilised in the pre-fusion conformation (K986P and V987P)^22^ with the furin-cleavage site ([PH]RRAR) either intact (‘2P’ constructs) or mutated to the uncleavable sequence PSRAS (‘FUR2P’ constructs). The following variant spikes were made (SA^20^, Alpha^19^ and mink^34^) (all mutations listed with reference to the NCBI sequence YP_009724390.1): 2P Alpha (Δ69-70, Δ144, N501Y, A570D, D614G, P681H, T716A, S982A, D1118H, and K986P, V987P), FUR2P Alpha (Δ69-70, Δ144, N501Y, A570D, D614G, T716A, S982A, D1118H, and R682S, R685S, K986P, V987P), FUR2P D614 Alpha (Δ69-70, Δ144, N501Y, A570D, T716A, S982A, D1118H, and R682S, R685S, K986P, V987P), 2P P681H-only (P681H and K986P, V987P), P681R-only (P681R and K986P, V987P), 2P Beta spike (L18F, D80A, D215G, R246I, K417N, E484K, N501Y, D614G, A701V, and K986P, V987P), FUR2P Beta spike (L18F, D80A, D215G, Δ242-244, R246I, K417N, E484K, N501Y, D614G, A701V, and R682S, R685S, K986P, V987P), FUR2P mink (Δ69-70, Y453F, D614G, I692V, and R682S, R685S, K986P, V987P), FUR2P D614 mink (Δ69-70, Y453F, I692V, and R682S, R685S, K986P, V987P). The Wuhan spikes and ACE2 ectodomain construct (residues 19-615) used in this study were exactly as described before^5,8^.

### Protein expression and purification

All the variant spikes were expressed in suspension-cultured Expi293F cells (Gibco) cells and purified as described before for the D614G spike^12^. In brief, Expi293F cells were cultured at 37 °C, shaking at 125 rpm, in humidified 8% CO_2_ atmosphere in FreeStyle 293 Expression Medium and transfected with 1 mg of spike DNA per litre of culture at cell density of 3 × 10^6^/mL. For expression in presence of a furin inhibitor, decanoyl-Arg-Val-Lys-Arg-chloromethylketone (also known as “furin inhibitor I”) was prepared as a 23.5 mg/mL stock solution in DMSO and added to the cells at final concentration of 100 µM half an hour prior to transfection. Next day after transfection, the cells were enhanced according to the manufacturer’s instructions and transferred to 32 °C^35^.

The supernatant was harvested on the fifth day post transfection and the spike purified using cobalt NTA beads (TAKARA). The protein was then eluted in PBS with 200 mM imidazole, concentrated, and either flash-frozen or gel filtered (Supplementary Fig. 2e) at room temperature into a buffer containing 150 mM NaCl, TRIS pH 8 on a Superdex 200 Increase 10/300 GL column (GE Life Sciences). Wuhan spikes and ACE2 were made exactly as described before^5,8^.

### Biolayer interferometry

Measurements of spike variants affinity towards human ACE2 ectodomain were performed at 25*°*C on an Octet Red 96 instrument (ForteBio) with shaking at 1000 rpm in 150 mM NaCl, 20 mM TRIS pH 8. First, variant spikes at 40–80 µg/mL were immobilised for 40–60 min on NiNTA sensors pre-equilibrated in buffer. Then, the ACE2 binding was measured using 2–5 min association and 10–20 min dissociation phases. At least three independent measurements were made for each spike.

The data were analysed using kinetic and equilibrium methods. For equilibrium analysis, the data were first normalised by dividing by the maximum observable response in order to give fractional saturation as a function of ACE2 concentration. The *K*_D_ was then determined from analysis of the variation of fractional saturation with ACE2 concentration. For kinetic analysis, plots of the observed rate (*k*_obs_) were derived from association phases using a single exponential function and *k*_on_ and *k*_off_ were obtained from plots of *k*_obs_ vs ACE2 concentration as the slope and intercept respectively.

### CryoEM sample preparation

Samples were frozen on R2/2 400 mesh Quantifoil grids glow-discharged for 30 s at 25 mA. A sample at 0.5–0.8 mg/mL final concentration of the spike was supplemented with 0.1% (final concentration) octyl glucoside, 4 µL of it applied on a grid, blotted for 5 s to 6 s with filter paper pre-equilibrated at 4 °C in 100% humidity, and plunge frozen in liquid ethane using Vitrobot Mark III. In order to obtain ACE2-spike complexes the proteins were mixed at 2:1 molar ratio of ACE2 to spike trimer and incubated at room temperature for 20–40 min prior to grid freezing.

### CryoEM data collection

Data were collected using EPU software (Thermo Scientific) on Titan Krios microscopes operating at 300 kV either with a Falcon 3 camera (Thermo Scientific) operating in electron-counting mode (60 s exposures, total dose of 35 e^−^/Å^2^, fractionated into 30 frames, 1.09 Å^2^ calibrated pixel size) or K2 camera (Gatan) with GIF Quantum LS energy filter (Gatan) with a slit width of 20 eV operating in the zero-loss mode (9.4 s exposures, 49 e^−^/Å^2^ total dose, fractionated into 32 frames, 1.08 Å^2^ calibrated pixel size). The following eight datasets were collected (Supplementary Table 1): FUR2P Beta (Falcon 3), ACE2 + FUR2P Beta (K2), FUR2P Alpha (Falcon 3), ACE2 + FUR2P D614 Alpha (K2), ACE2 + 2P Alpha furin-uninhibited (Falcon 3), ACE2 + 2P Alpha furin-inhibited (Falcon 3), FUR2P mink (K2), ACE2 + FUR2P D614 mink (K2). All micrographs were collected with defocus range between 1.5 and 3 µm.

### CryoEM data processing

Collected movies were motion corrected using MotionCor2^36^ implemented in RELION^37^ and contrast transfer functions estimated using CTFFind4^38^. Particles were picked using crYOLO^39^ using manually trained models. Particles were extracted 2x downsampled in RELION before two rounds 2D classification in cryoSPARC^40^. Classes that showed clear secondary structure were retained and an initial model generated also using cryoSPARC. Particles from the selected classes were 3D classified in RELION. The selection process for the different data collections are shown in Supplementary Figs. 3, 4, 6. Final particle stacks were re-extracted unbinned and subjected to Bayesian polishing in RELION, and refined in cryoSPARC using either Homogeneous refinement or Non-Uniform Refinement routines, both coupled to per-particle defocus refinement. Final maps had their local resolution estimated using blocres^41^ implemented in cryoSPARC (Supplementary Fig. 8), followed by local resolution filtering in cryoSPARC and B-factor sharpening^42^.

### Model building, refinement, and validation

High-resolution models of the monomeric S-ACE2 complexes were based on the previously determined model for the non-uniform map refinement of the monomeric Wuhan spike in complex with ACE2 (PDB 7A91)^5^. The low-resolution model of the monomeric Alpha S-ACE2 complex was built from the non-uniform-refined, high resolution model of the same protein from this study combined with the monomeric Wuhan S-ACE2 (PDB 7A92)^5^. Models for the unbound trimeric spikes were based on our model of D614G spike (PDB IDs 7BNM, 7BNN, 7BNO)^12^. The model of the three-ACE2-bound trimer of the 2P Alpha spike was based on the three-ACE2-bound Wuhan spike we determined before (PDB ID 7A98)^5^, in which the RBD (spike residues 333-527) and ACE2 were replaced by those from the monomeric, non-uniformed-refined Alpha S-ACE2 determined in this study. All structures were manually adjusted in COOT^43^, refined with PHENIX Real Space Refine and validated in PHENIX^44^. Measurements were performed with CCP4mg^45^ and Chimera^46^.

### Reporting summary

Further information on research design is available in the Nature Research Reporting Summary linked to this article.

## Supplementary information

Supplementary Information

Reporting Summary

## Data Availability

The data that support this work is available from the corresponding author upon reasonable request. The cryoEM maps and models generated in this study have been deposited in the Electron Microscopy Data Bank with accession numbers EMD-14225, EMD-14226, EMD-14227, EMD-14228, EMD-14229, EMD-14230, EMD-14231, EMD-14232, EMD-14233, EMD-14234, EMD-14237, EMD-14235, EMD-14236. Models have been deposited in the Protein Data Bank, https://www.ebi.ac.uk/pdbe/ (PDB ID codes 7R0Z, 7R10, 7R11, 7R12, 7R13, 7R14, 7R15, 7R16, 7R17, 7R18, 7R1B, 7R19, 7R1A). Source data are provided with this paper.

## Source data

Source Data
