# Supplementary material for: Evolution of the SARS-CoV-2 spike protein in the human host
Source: Nat Commun. 2022 Mar 4;13:1178. doi: 10.1038/s41467-022-28768-w (PMC8897445; doi:10.1038/s41467-022-28768-w)
Supplement: Supplementary file 1 — Supplementary Information [file 41467_2022_28768_MOESM1_ESM.pdf]

Supplementary Information

Evolution of the SARS-CoV-2 spike protein in the human host

Wrobel, Benton, et al., *Nature Communications 2022*

Alpha Variant

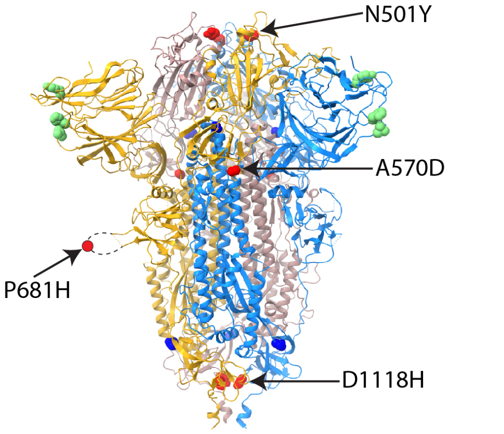

Alpha Variant Changes:

del69-70
del144
N501Y
A570D
P681H
T716I
S982A
D1118H

Beta Variant

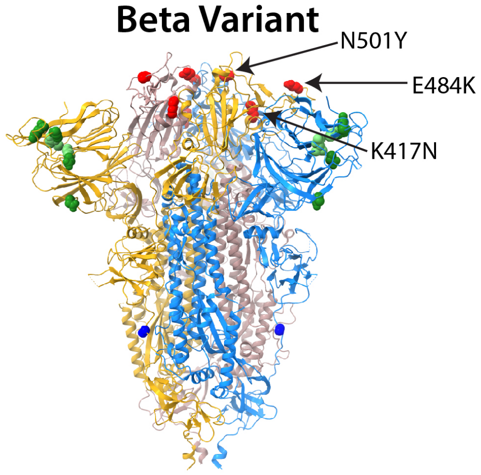

Beta Variant Changes:

L18F
D80A
D215G
del242-244
R246I
K417N
E484K
N501Y
A701V

Mink Variant

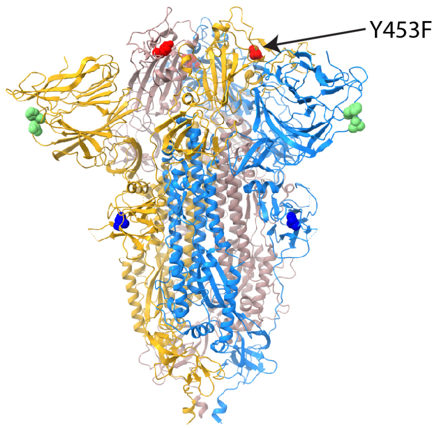

Mink Variant Changes:

del69-70
Y453F
I692V

Supplementary Figure 1: Location of changes in spike protein of different SARS-CoV-2 variants investigated in this study. Changes are highlighted on our previous structure of SARS-CoV-2 spike in closed conformation (PDB ID 6ZGE[8]) (left). Substitutions of particular interest–located either on the intra-trimer interfaces or the RBD–are highlighted in red. Changes in the NTD–mainly restricted to surface interfaces–are shown in either dark (substitutions) or light (deletions) green. Other changes are highlighted in blue. A full list of changes is shown for each variant on the right.

Alpha spike, furin uninhibited (cleaved)

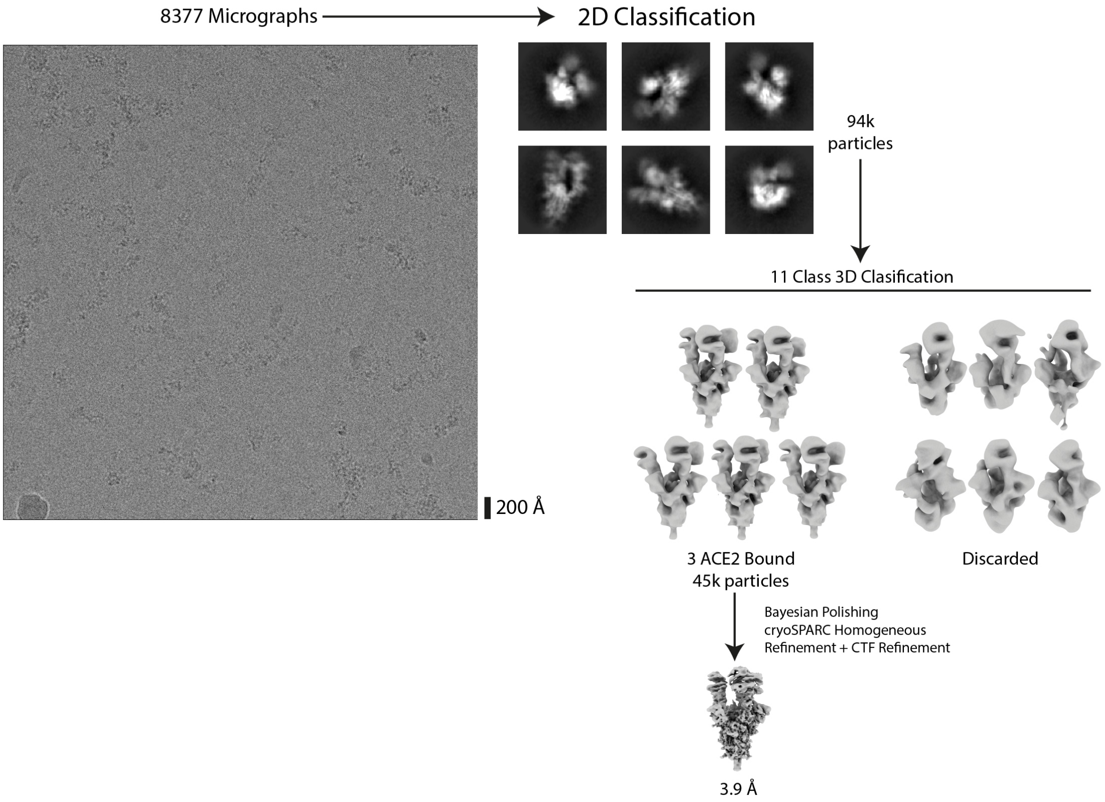

Alpha spike, furin inhibited (uncleaved)

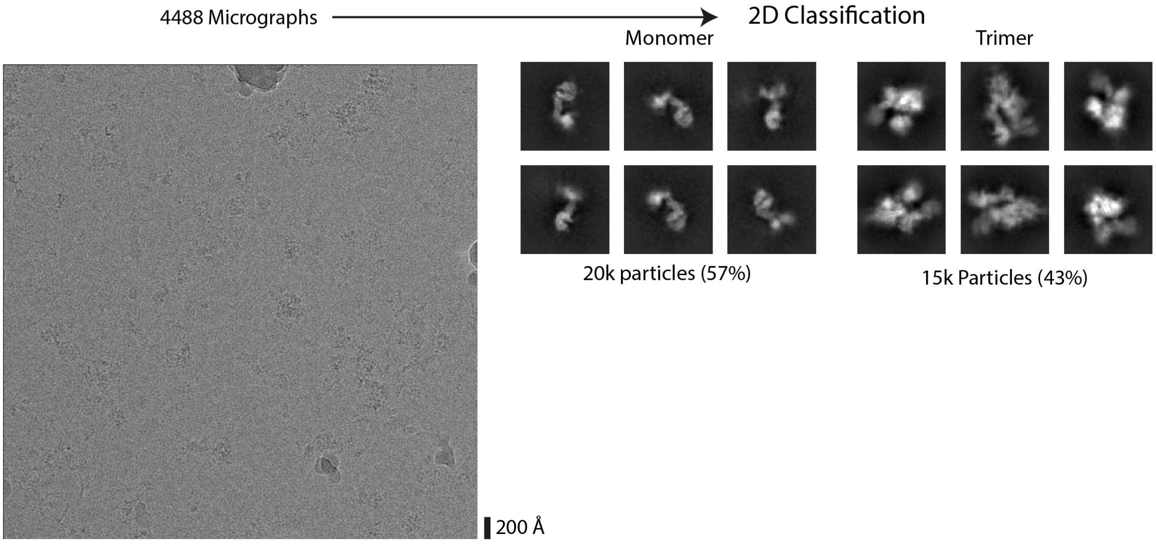

Supplementary Figure 2: cryoEM image processing scheme for Alpha variant in furin uninhibited (cleaved) and furin inhibited (uncleaved) forms binding to ACE2. Typical micrographs are shown on the left.

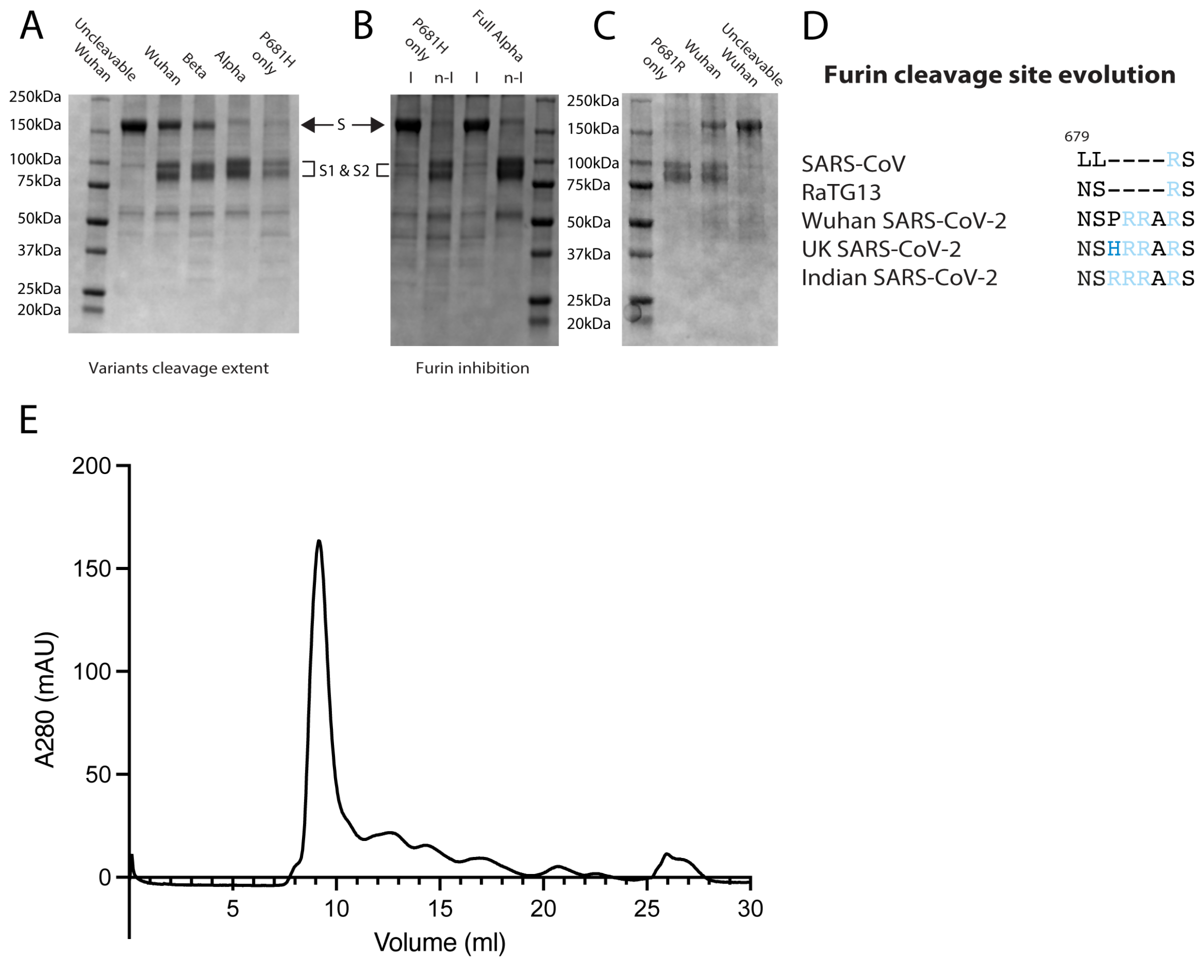

Supplementary Figure 3: Cleavage state of variant spikes. (A, B, C) SDS PAGE analysis of purified spikes used in this study. Each gel sample has been run at least three times with similar results. (A) From left, lanes contain: molecular weight markers (Precision Blue Protein Standards, All Blue, Bio-rad), uncleavable ('FUR2P': R682S+R685S) Wuhan spike, and 2P-only spikes: Wuhan, SA, Alpha, P681H-only (otherwise identical to the Wuhan 2P). Alpha and P681H-only spikes are almost fully cleaved into S1 and S2 while Beta and Wuhan spikes are cleaved only partially; this indicates that the P681H substitution in the cleavage site is sufficient to cause the extent of cleavage observed in the Alpha variant. (B) Cleavage state of P681H-only (left two lanes) and Alpha (right two lanes) spikes expressed in presence of furin inhibitor I (lanes "I") compared to non-inhibited (n-I). The inhibitor almost fully prevents spike cleavage into S1 and S2. (C) Cleavage state of the P681R-only spike (left lane) compared to the Wuhan, furin-cleavable (middle lane) and uncleavable (right lane) spikes. The P681R, present in Delta and Kappa variants isolated recently in India, is sufficient to cause almost full cleavage of the spike into S1 and S2. (D) Sequence alignment of the S1/S2 cleavage site from several SARS-CoV-2 strains and related sarbecoviruses: SARS-CoV and the bat-CoV most closely related to SARS-CoV-2, RaTG13. SARS-CoV-2 acquired furin-cleavage site RxxR during its evolution and this site has become more polybasic (blue) in the recent Alpha and Indian variants. (E) A chromatogram (of cleaved Alpha spike) from size exclusion chromatography representative for the spikes used in this study. Source data are provided as a Source Data file.

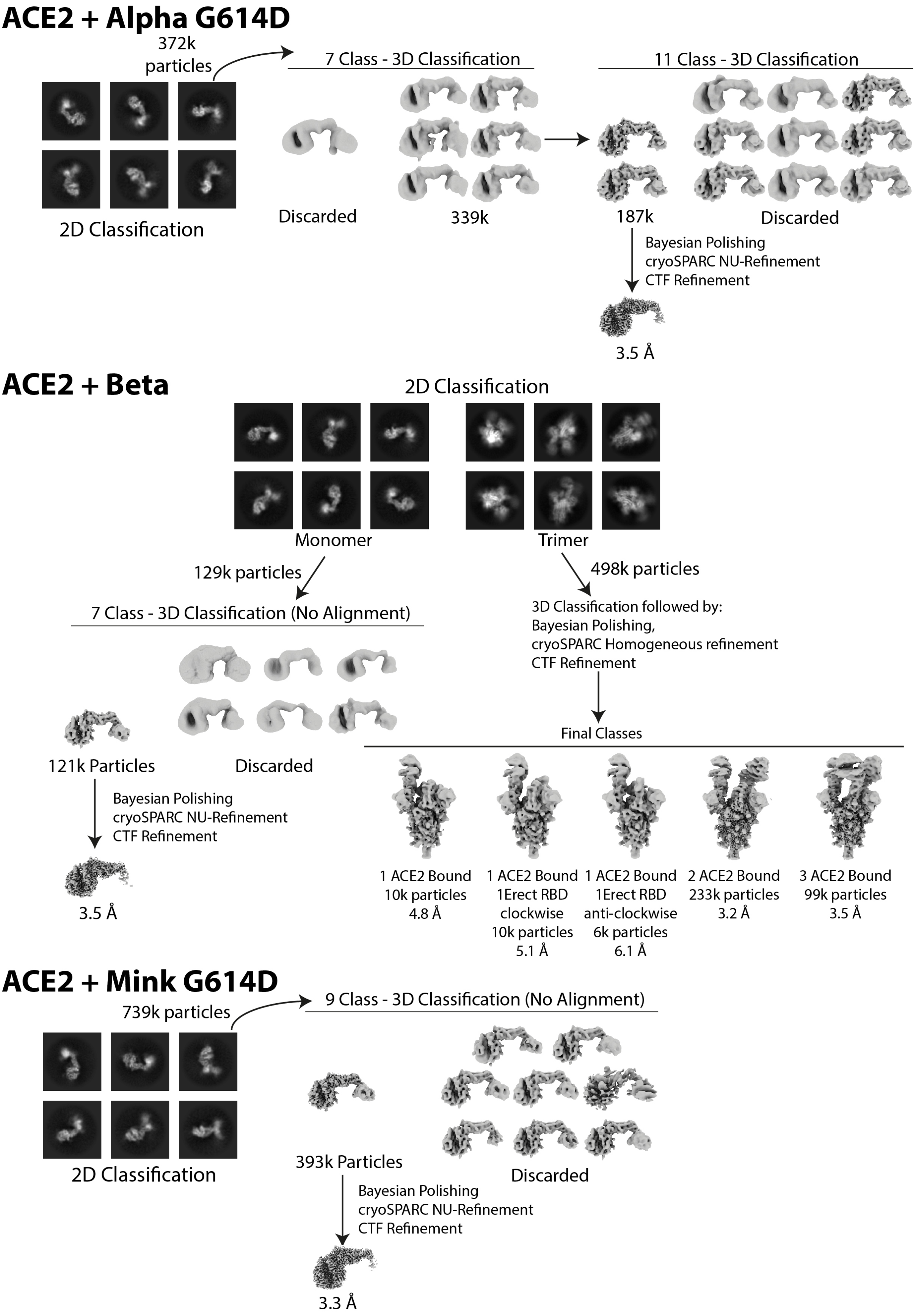

Supplementary Figure 4: cryoEM image processing scheme for Alpha (G614D), Beta and Mink (G614D) variants in complex with ACE2.

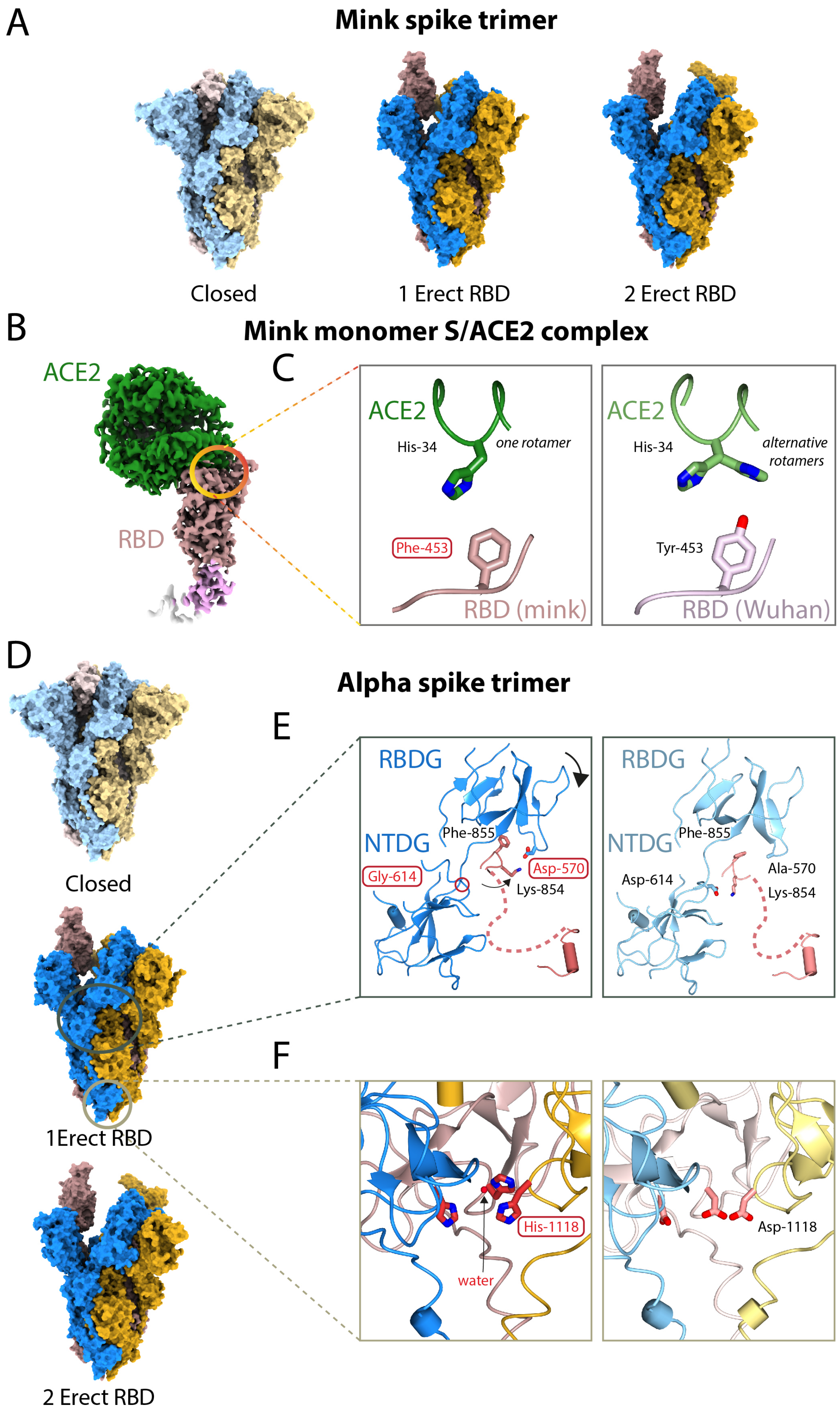
A
Mink spike trimer
Closed
1 Erect RBD
2 Erect RBD
B
Mink monomer S/ACE2 complex
ACE2
RBD
C
ACE2
His-34
one rotamer
Phe-453
RBD (mink)
ACE2
His-34
alternative rotamers
Tyr-453
RBD (Wuhan)
D
Closed
1Erect RBD
2 Erect RBD
Alpha spike trimer
E
RBDG
NTDG
Phe-855
Gly-614
Asp-570
Lys-854
RBDG
NTDG
Phe-855
Asp-614
Ala-570
Lys-854
F
His-1118
water
Asp-1118

Supplementary Figure 5: Structural insights into mink and Alpha variant spikes. (A) Surface representations of mink spike structures determined in this study: in a dilated, closed conformation different to that of the Wuhan spike but resembling D614G spike[12]; and one- and two-RBD erect conformations, the latter of which is assumed by D614G but not by Wuhan spike[12]. (B) Cryo-EM density of monomeric complex of D614 mink spike and ACE2. (C) Detail of the mink spike / ACE2 interface (left) compared to Wuhan (right). The His-34, which adopts two alternative conformations in Wuhan spike, is present only as one rotamer in the structure of the mink spike/ACE2 complex (Fig. S4).

(D) Surface representations of furin-uncleavable Alpha spike in three conformations which resemble those of the mink and D614G-only spike we described previously[12]: a dilated-closed conformation different to that of the Wuhan variant and two open conformations, with one or two RBDs erect.

(E, F) Detail of substitutions in Alpha spike (left panels) compared to Wuhan (right panels) that may further contribute to enhanced trimeric state of the open and receptor-bound forms of Alpha spike. (E) A570 in Wuhan strain lies close to the interface between RBD-associated subdomain (RBDG, blue) and the fragment of S2 core of the neighbouring chain (residues 815-855, red), which undergoes significant rearrangement upon spike opening (the unfolded region 824-853 is structured in the closed Wuhan spike). In (D570) Alpha spike the whole RBDG domain shifts slightly closer towards S2 and the same region of the neighbouring-chain S2, especially F855, adopts different conformation upon spike opening. This alternative conformation might be attributed to a formation of a salt bridge between K854, which incidentally makes a salt bridge with D614 in the original Wuhan strain but not in the later G614 variants, and D570 in the Alpha variant spike. (F) The S2 residue 1118 lies at the membrane proximal end of the Alpha spike trimer, close to the trimer axis. It is an aspartic acid in Wuhan (right) but a histidine in Alpha. It appears that a cluster of trimer-related aspartic acid side chains at this position forms a less stable arrangement than is be adopted by a corresponding cluster of neutral histidine residues in the Alpha spike, which also coordinate a smaller molecule or an ion–here modelled as water. These two substitutions could explain greater stability of the open form of Alpha spike. Indeed, a comparison of the overall surface areas of the monomer-monomer interfaces in the 1-RBD-erect conformations of the Alpha and G614-only trimers shows that the former is 700 Å^2 larger providing more evidence to why the Alpha spike is less likely to undergo disassembly upon receptor binding than other variants.

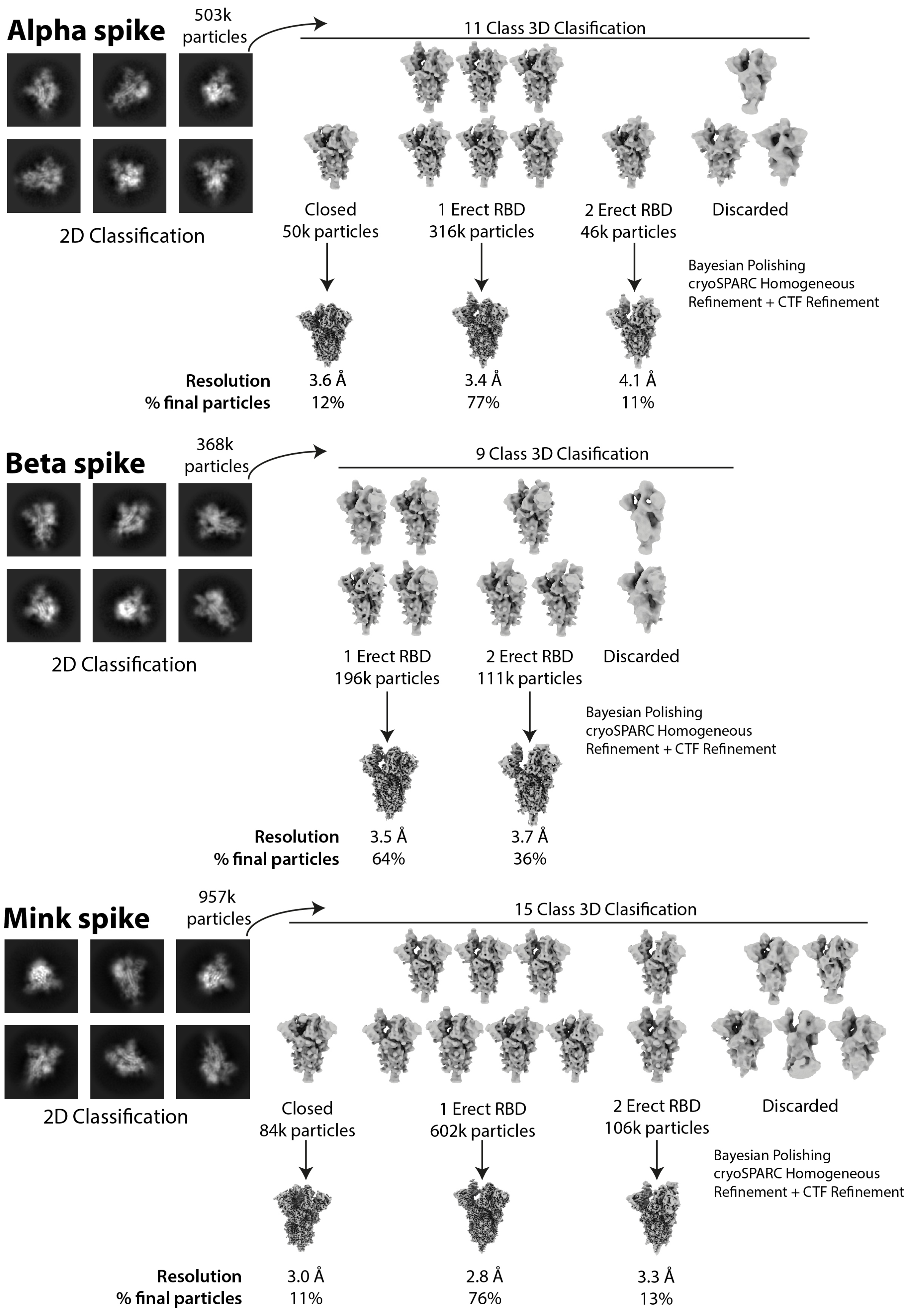

Supplementary Figure 6: cryoEM image processing scheme for spike trimers of Alpha, Beta and Mink variants.

A

Spike	kon ($\mu M^{-1}s^{-1}$)	koff (s^{-1})	K_d (Kin) (nM)	K_d (Amp) (nM)
Wuhan *	0.119 ± 0.005	0.0107 ± 0.0015	90.7 ± 14.3	114.2 ± 17.5
Alpha G614D	0.130 ± 0.006	0.0124 ± 0.0017	93.0 ± 14.0	111.4 ± 21.9
Mink G614D	0.103 ± 0.004	0.0112 ± 0.0015	113.0 ± 15.3	90.5 ± 22.4
Wuhan D614G	0.075 ± 0.003	0.0108 ± 0.0014	144.0 ± 19.0	154.2 ± 29.7
Alpha	0.138 ± 0.003	0.0023 ± 0.0012	16.7 ± 7.6	25.3 ± 4.2
Mink	0.088 ± 0.006	0.0033 ± 0.0014	36.6 ± 10.5	46.2 ± 5.8
Beta	0.180 ± 0.004	0.0075 ± 0.0019	44.7 ± 10.5	31.2 ± 4.3

B

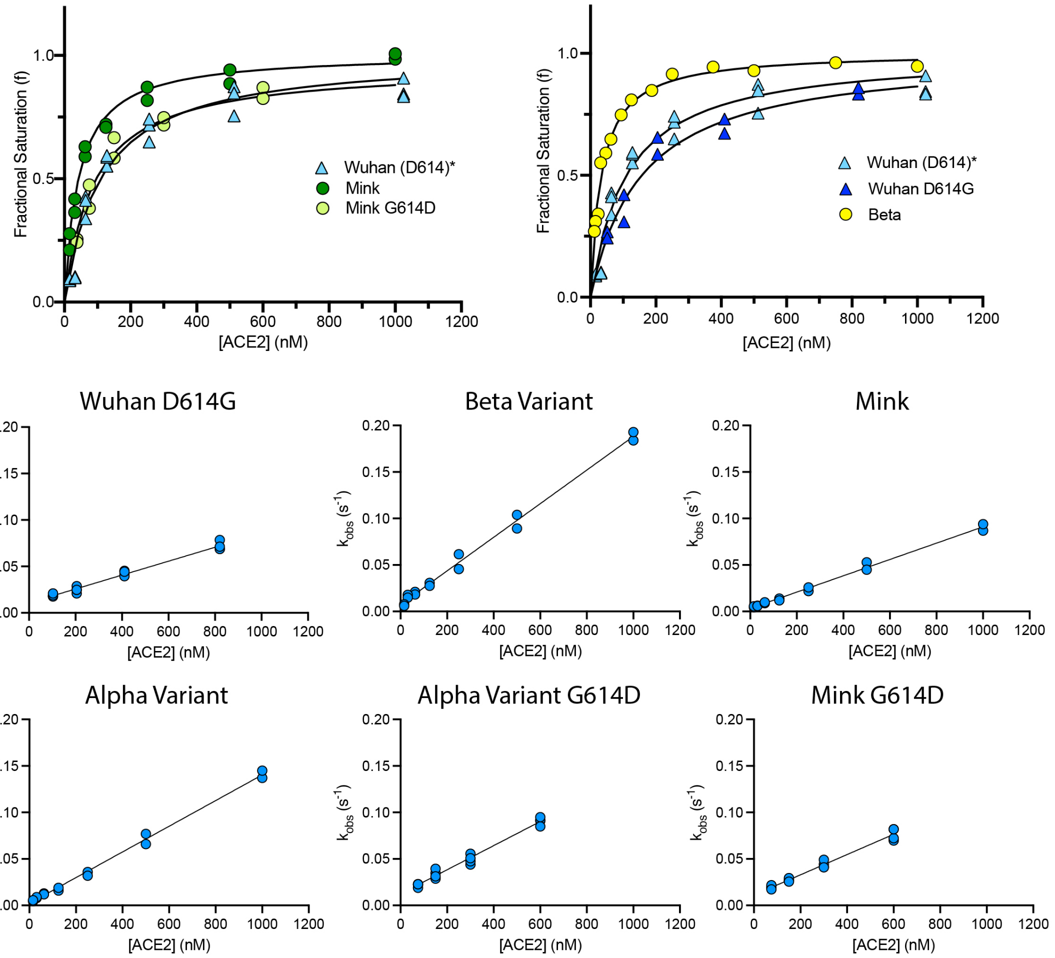

Supplementary Figure 7: Biolayer interferometry binding measurements of ACE2 binding to immobilised variant spikes. (A) Thermodynamic parameters for ACE2 binding to different spikes. Association rate constants (k_{on}) were determined from the slopes of plots of the observed rate constant against ACE2 concentration shown in panel C. Dissociation rate constants (k_{off}) were determined from the intercepts of these plots and through independent analysis of the dissociation phase. The K_d values were calculated from the kinetic data as k_{off}/k_{on} (K_d(Kin)) and from analysis of the dependence of fractional saturation on ACE2 concentration shown in main Fig. 2b and panel B below (K_d(Amp)). (B) Variation of fractional saturation with ACE2 concentration for different spikes. The solid lines are the computed best fits. * The data for Wuhan (D614) shown here are adapted from our previous work[48]. (C) Dependence of the observed rate constant (k_{obs}) on ACE2 concentration for different spikes. Source data are provided as a Source Data file.

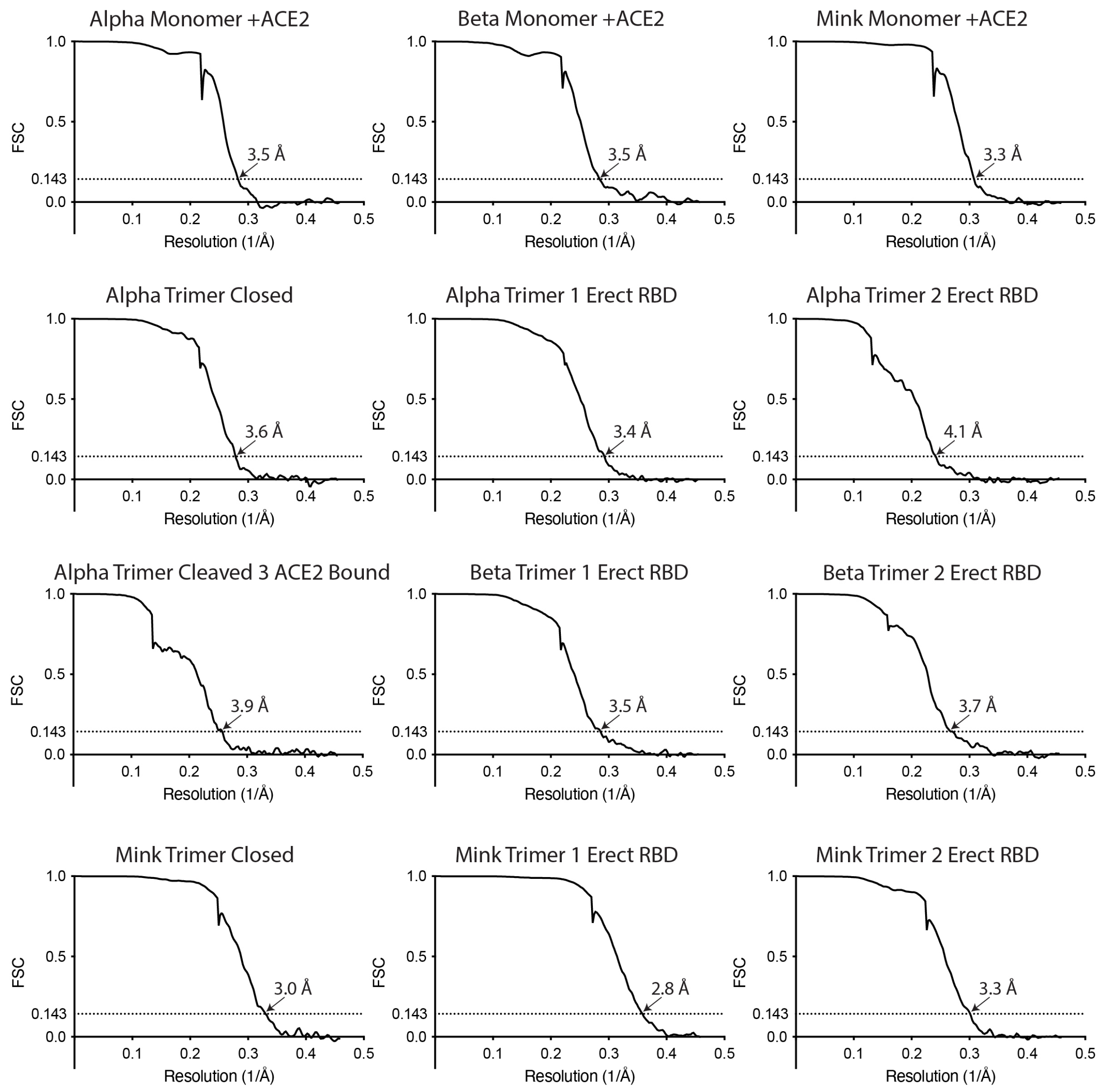

Figure S8: Fourier Shell Correlation (FSC) curves for each of the deposited structures.

Supplementary Table 1:

Cryo-EM data collection, refinement and validation statistics

	Alpha Monomer +ACE2 (EMDB-14225) (PDB 7R0Z)	Alpha Monomer +ACE2 (with accessories) (EMDB-14226) (PDB 7R10)	Beta Monomer +ACE2 (EMDB-14427) (PDB 7R11)	Mink Monomer +ACE2 (EMDB-14228) (PDB 7R12)	Alpha Trimer Closed (EMDB-14229) (PDB 7R13)	Alpha Trimer 1 Erect RBD (EMDB-14230) (PDB 7R14)	Alpha Trimer 2 Erect RBD (EMDB-14231) (PDB 7R15)	Beta Trimer 1 Erect RBD (EMDB-14232) (PDB 7R16)	Beta Trimer 2 Erect RBD (EMDB-14233) (PDB 7R17)	Mink Trimer Closed (EMDB-14234) (PDB 7R18)	Mink Trimer 1 Erect RBD (EMDB-14237) (PDB 7R1B)	Mink Trimer 2 Erect RBD (EMDB-14235) (PDB 7R19)	Alpha Trimer (cleaved) + 3 ACE2 (EMDB-14236) (PDB 7R1A)
**Data collection and processing**													
Voltage (kV)	300	300	300	300	300	300	300	300	300	300	300	300	300
Electron exposure (e–/Å^2)	49	49	49	49	35	35	35	35	35	49	49	49	35
Defocus range (μm)	-1.5 to -3.0	-1.5 to -3.0	-1.5 to -3.0	-1.5 to -3.0	-1.5 to -3.0	-1.5 to -3.0	-1.5 to -3.0	-1.5 to -3.0	-1.5 to -3.0	-1.5 to -3.0	-1.5 to -3.0	-1.5 to -3.0	-1.5 to -3.0
Pixel size (Å)	1.08	1.08	1.08	1.08	1.09	1.09	1.09	1.09	1.09	1.08	1.08	1.08	1.09
Symmetry imposed	C1	C1	C1	C1	C3	C1	C1	C1	C1	C3	C1	C1	C3
Final particle images (no.)	187k	187k	121k	393k	50k	316k	46k	196k	111k	84k	602k	106k	45k
Map resolution (Å)<br>FSC threshold = 0.143	3.5	4.0	3.5	3.3	3.6	3.4	4.1	3.5	3.7	3.0	2.8	3.3	3.9
**Refinement**													
Initial model used (PDB code)	7A91	7A92	7A91	7A91	7BNM	7BNN	7BNO	7BNN	7BNO	7BNM	7BNN	7BNO	7A98
Model resolution (Å)<br>FSC threshold = 0.5	3.5	4.0	3.7	3.3	3.6	3.4	4.3	3.6	3.9	3.2	2.9	3.5	4.0
Map sharpening *B* factor (Å^2)	-130.2	-108.6	-132.7	-134.2	-140.0	-151.5	-123.0	-141.7	-132.8	-98.7	-103.0	-88.4	-119.0
Model composition													
Non-hydrogen atoms	6891	9823	6870	6858	24303	23655	24109	24067	23641	25773	25562	25400	38670
Protein residues	839	1210	837	839	3003	2939	3000	3005	2981	3189	3193	3178	4758
Ligands (Glycans)	9	8	8	7	57	50	43	40	22	60	42	38	60
R.m.s. deviations													
Bond lengths (Å)	0.005	0.002	0.003	0.003	0.003	0.005	0.004	0.004	0.003	0.005	0.004	0.003	0.003
Bond angles (°)	0.621	0.457	0.529	0.529	0.547	0.607	0.526	0.571	0.504	0.673	0.651	0.556	0.511
Validation													
MolProbity score	1.27	1.53	1.42	1.33	1.67	1.78	1.97	1.60	1.46	1.32	1.34	1.21	1.76
Clashscore	3.78	6.34	4.17	4.03	6.22	5.29	10.95	4.65	5.15	2.68	3.31	3.99	8.24
Poor rotamers (%)	0.00	0.00	0.54	0.00	0.00	0.04	0.00	0.19	0.50	0.32	0.54	0.18	0.00
Ramachandran plot													
Favored (%)	97.47	96.96	96.62	97.23	95.32	91.81	93.85	94.80	96.88	96.07	96.64	97.90	95.50
Allowed (%)	2.53	3.04	3.38	2.77	4.68	8.19	6.15	5.20	3.12	3.93	3.36	2.10	4.50
Disallowed (%)	0.00	0.00	0.00	0.00	0.00	0.00	0.00	0.00	0.00	0.00	0.00	0.00	0.00
