# Supplementary material for: Evolution of the SARS-CoV-2 spike protein in the human host
Source: Nat Commun. 2022 Mar 4;13:1178. doi: 10.1038/s41467-022-28768-w (PMC8897445; doi:10.1038/s41467-022-28768-w)
Supplement: Supplementary file 2 — Reporting Summary [file 41467_2022_28768_MOESM2_ESM.pdf]

Corresponding author(s): Antoni Wrobel, Donald Benton, Steven Gamblin

Last updated by author(s): Feb 4, 2022

Reporting Summary

Nature Portfolio wishes to improve the reproducibility of the work that we publish. This form provides structure for consistency and transparency in reporting. For further information on Nature Portfolio policies, see our Editorial Policies and the Editorial Policy Checklist.

Statistics

For all statistical analyses, confirm that the following items are present in the figure legend, table legend, main text, or Methods section.

n/a	Confirmed	
☐	☒	The exact sample size (n) for each experimental group/condition, given as a discrete number and unit of measurement
☒	☐	A statement on whether measurements were taken from distinct samples or whether the same sample was measured repeatedly
☒	☐	The statistical test(s) used AND whether they are one- or two-sided<br>*Only common tests should be described solely by name; describe more complex techniques in the Methods section.*
☒	☐	A description of all covariates tested
☒	☐	A description of any assumptions or corrections, such as tests of normality and adjustment for multiple comparisons
☒	☐	A full description of the statistical parameters including central tendency (e.g. means) or other basic estimates (e.g. regression coefficient) AND variation (e.g. standard deviation) or associated estimates of uncertainty (e.g. confidence intervals)
☒	☐	For null hypothesis testing, the test statistic (e.g. F, t, r) with confidence intervals, effect sizes, degrees of freedom and P value noted<br>*Give P values as exact values whenever suitable.*
☒	☐	For Bayesian analysis, information on the choice of priors and Markov chain Monte Carlo settings
☒	☐	For hierarchical and complex designs, identification of the appropriate level for tests and full reporting of outcomes
☒	☐	Estimates of effect sizes (e.g. Cohen's d, Pearson's r), indicating how they were calculated

Our web collection on statistics for biologists contains articles on many of the points above.

Software and code

Policy information about availability of computer code

Data collection	CryoEM data collected using Thermo Scientific EPU v2.9
Data analysis	CryoEM data processed using following packages: RELION-3.1, cryoSPARC v3.2, CTFfind4 v.4.1.10, MotionCor2 v.1.2.6, crYOLO v1.4, Coot v.0.9, PHENIX v.1.17, UCSF Chimera v.1.12, CCP4MG v2.10

For manuscripts utilizing custom algorithms or software that are central to the research but not yet described in published literature, software must be made available to editors and reviewers. We strongly encourage code deposition in a community repository (e.g. GitHub). See the Nature Portfolio guidelines for submitting code & software for further information.

Data

Policy information about availability of data

All manuscripts must include a data availability statement. This statement should provide the following information, where applicable:

- Accession codes, unique identifiers, or web links for publicly available datasets
- A description of any restrictions on data availability
- For clinical datasets or third party data, please ensure that the statement adheres to our policy

The cryoEM maps and models generated in this study have been deposited in the Electron Microscopy Data Bank, http://www.ebi.ac.uk/pdbe/emdb/ (Accession numbers EMD-14225, EMD-14226, EMD-14227, EMD-14228, EMD-14229, EMD-14230, EMD-14231, EMD-14232, EMD-14233, EMD-14234, EMD-14237, EMD-14235, EMD-14236). Models have been deposited in the Protein Data Bank, https://www.ebi.ac.uk/pdbe/ (PDB ID codes 7R0Z, 7R10, 7R11, 7R12, 7R13, 7R14, 7R15, 7R16, 7R17, 7R18, 7R1B, 7R19, 7R1A).

Field-specific reporting

Please select the one below that is the best fit for your research. If you are not sure, read the appropriate sections before making your selection.

[x] Life sciences [ ] Behavioural & social sciences [ ] Ecological, evolutionary & environmental sciences

For a reference copy of the document with all sections, see nature.com/documents/nr-reporting-summary-flat.pdf

Life sciences study design

All studies must disclose on these points even when the disclosure is negative.

Sample size	The cryoEM dataset we collected consisted of thousands of images. The reported number of images (Table S1) for each data set was sufficient to achieve the final resolution calculated according to the most commonly used resolution measure in cryoEM described in Rosenthal and Henderson, 2003 (cited in the manuscript).
Data exclusions	cryoEM data were excluded and included according to the standard image processing work-flow employing established image processing techniques such as 2D and 3D classifications, as detailed in Supplementary Figures S3, S4, S6, and S8.
Replication	All biophysical experiments were repeated three times or more, with no unsuccessful replications, and with similar results
Randomization	Randomisation was not applicable to this study, as there were no experimental groups to assign the samples to, and data were processed according to standard cryoEM procedures.
Blinding	As there was no assignment of experimental groups in data collection or analysis, the blinding was not applicable to this study.

Reporting for specific materials, systems and methods

We require information from authors about some types of materials, experimental systems and methods used in many studies. Here, indicate whether each material, system or method listed is relevant to your study. If you are not sure if a list item applies to your research, read the appropriate section before selecting a response.

Materials & experimental systems

n/a	Involved in the study
[x]	[ ] Antibodies
[ ]	[x] Eukaryotic cell lines
[x]	[ ] Palaeontology and archaeology
[x]	[ ] Animals and other organisms
[x]	[ ] Human research participants
[x]	[ ] Clinical data
[x]	[ ] Dual use research of concern

Methods

n/a	Involved in the study
[x]	[ ] ChIP-seq
[x]	[ ] Flow cytometry
[x]	[ ] MRI-based neuroimaging

Eukaryotic cell lines

Policy information about cell lines

Cell line source(s)	expi293F cells were purchased from Thermo Scientific and used for protein expression
Authentication	Cell line used was not authenticated, although it came from a commercial source
Mycoplasma contamination	Cell line was not tested for mycoplasma contamination
Commonly misidentified lines (See ICLAC register)	No commonly misidentified cell lines were used in this study

March 2021
