# Supplementary figures and images for: Evolution of the SARS-CoV-2 spike protein in the human host
Source: Nat Commun. 2022 Mar 4;13:1178. doi: 10.1038/s41467-022-28768-w (PMC8897445; doi:10.1038/s41467-022-28768-w)

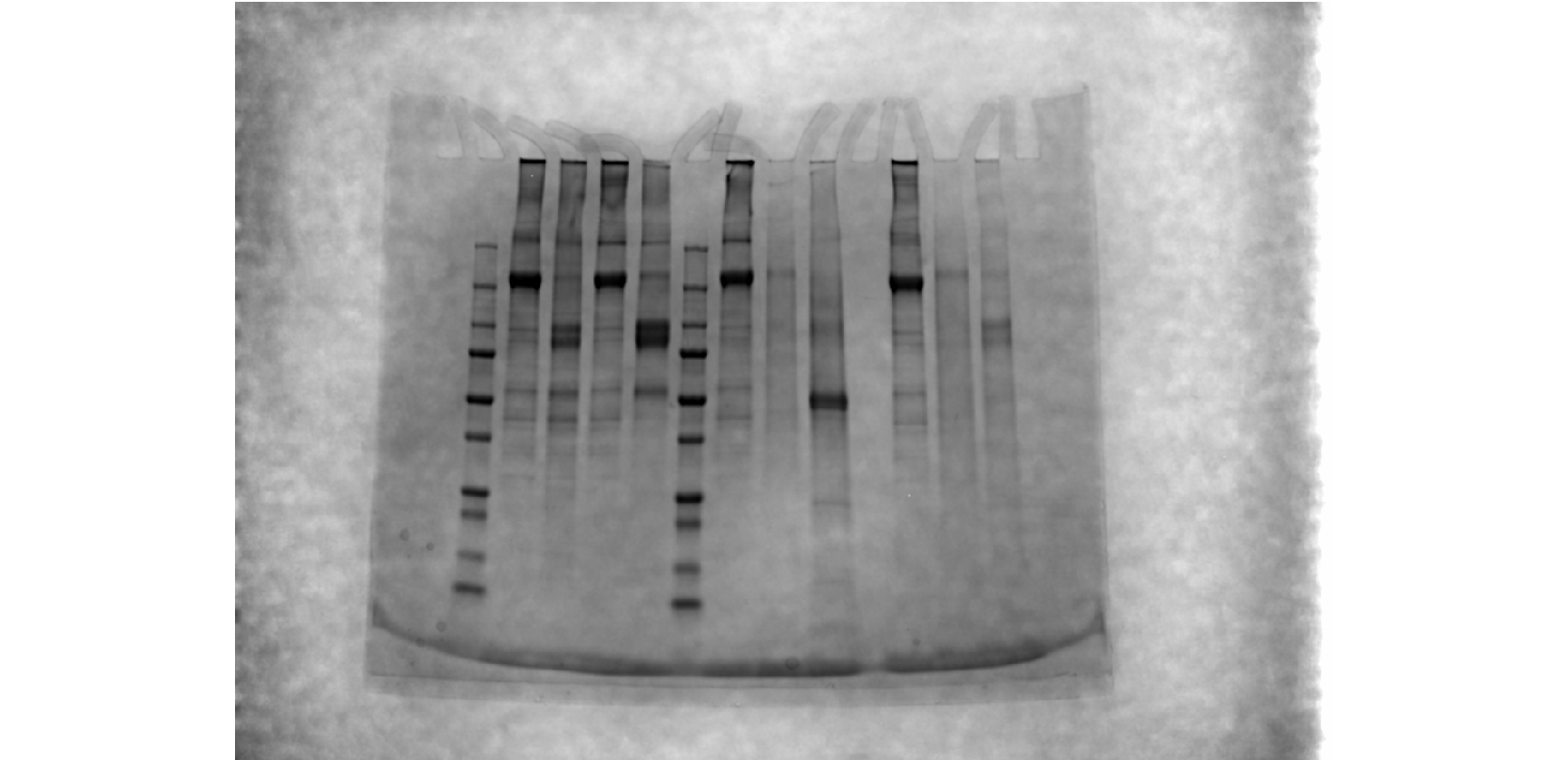

Supplement: Supplementary file 3 — Source Data [file 41467_2022_28768_MOESM3_ESM.zip › Source-data_3_gelS3b.tif]

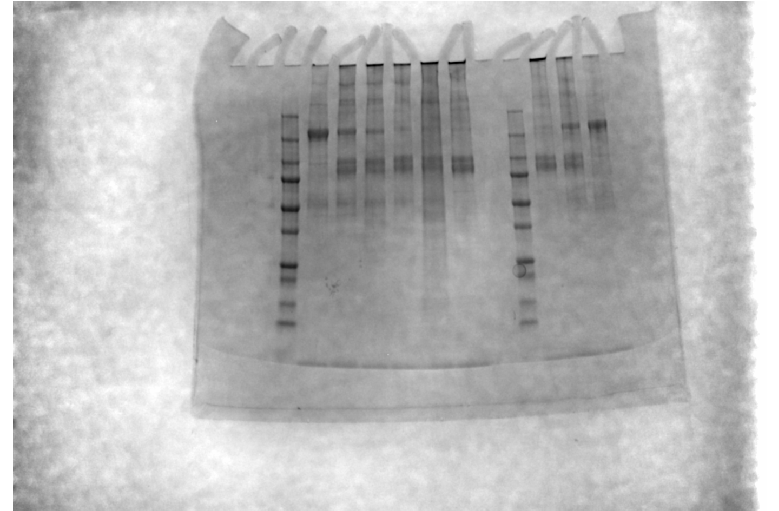

Supplement: Supplementary file 3 — Source Data [file 41467_2022_28768_MOESM3_ESM.zip › Source-data_4_gelS3c.tif]

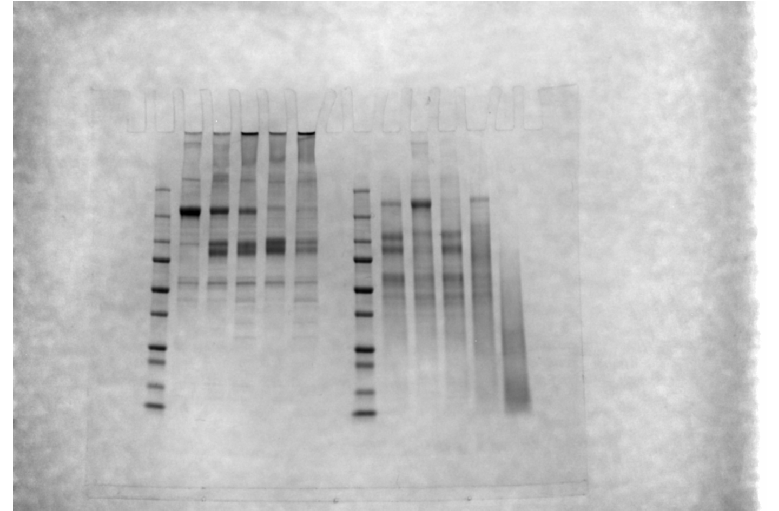

Supplement: Supplementary file 3 — Source Data [file 41467_2022_28768_MOESM3_ESM.zip › Source-data_2_gelS3a.tif]
